# Baseline factors affecting diabetic macular oedema resolution after intravitreal dexamethasone implant treatment: post hoc analysis of the MEAD study

**DOI:** 10.1186/s12886-025-04208-3

**Published:** 2025-07-09

**Authors:** Carolina C. S. Valentim, Hongxin Lai, Miller J. Ogidigben, Rishi P. Singh, Katherine E. Talcott

**Affiliations:** 1 Center for Ophthalmic Bioinformatics, Cole Eye Institute, Cleveland Clinic Foundation, 9500 Euclid Ave. i32, Cleveland, OH 44195 USA; 2 Kentucky Lions Eye Center, University of Louisville, Louisville, KY USA; 3 AbbVie Inc., North Chicago, IL USA

**Keywords:** Dexamethasone, Diabetic macular oedema, Diabetic retinopathy, MEAD study, OZURDEX, Pooled analysis, Predictive factors

## Abstract

**Background:**

Considering evidence that some baseline clinical parameters correlate with diabetic macular oedema (DME) response to intravitreal anti-VEGF treatment and prognosis, investigation of baseline characteristics that could predict treatment response to dexamethasone intravitreal implant (DEX) and facilitate treatment selection was warranted. This study evaluated the relationship between baseline characteristics and time to first DME resolution in patients treated with DEX.

**Methods:**

This post hoc analysis of the MEAD study (which consisted of 2 randomised, multicentre, masked, sham-controlled, phase 3 clinical trials identical in design) included 351 eyes treated with DEX 0.7 mg and 350 with sham over 3 years, with retreatment possible every ≥ 6 months if eligibility criteria were met. The effect of baseline characteristics on the time to first DME resolution (defined as central retinal/subfield thickness [CRT] < 250 μm) was evaluated with univariate and multivariate models, and further assessed with Kaplan-Meier method.

**Results:**

The median (95% CI) time to first DME resolution was 9.0 (8.5–9.3) months for the DEX group. The hazard ratio for DME resolution (DEX versus sham) was 2.09 (*P* < 0.0001). Higher CRT was associated with longer time to DME resolution.

**Conclusions:**

Treatment with DEX shortened the time to DME resolution compared with sham. Higher CRT was associated with longer time to DME resolution. These findings may influence therapeutic decisions.

**Trial registration:**

clinicaltrials.gov NCT00168337 and NCT00168389.

**Supplementary Information:**

The online version contains supplementary material available at 10.1186/s12886-025-04208-3.

## Introduction

Diabetes mellitus (DM) is prevalent in the United States, with ∼ 34.2 million people (all ages) diagnosed, in 2018, and an increasing prevalence [1, 2]. DM detection can take years, and its complications are common. Diabetic retinopathy (DR), the main ophthalmic complication of DM, is the leading cause of new cases of blindness among American adults [1]. The most common cause of vision loss in DR is diabetic macular oedema (DME), a build-up of fluid within the retina layers that can be quantitatively measured and monitored by optical coherence tomography (OCT) [3, 4].

Due to the chronic, progressive nature of DME, timely treatment is necessary. Intravitreal injections of anti-vascular endothelial growth factors (anti-VEGF) have been shown to efficiently treat DME and improve vision, and have supplanted laser photocoagulation as first-line treatment [5–10]. However, in cases of nonresponse and high treatment burden, intravitreal steroids offer an alternative option [11, 12].

The dexamethasone (DEX) intravitreal implant (Ozurdex; AbbVie Inc., North Chicago, IL, USA) is a sustained-release, biodegradable implant that releases DEX into the vitreous over ≤ 6 months [13]. Developed to reduce the need for frequent intraocular injections, it was approved as DME treatment in 2014, based on results of the phase 3 MEAD study, which consisted of 2 randomised, multicentre, masked, sham-controlled clinical trials identical in design [14]. In the pooled analysis of these 3-year trials, DEX implant resulted in a significantly greater percentage of patients with ≥ 15-letter improvements from baseline in best corrected visual acuity (BCVA) than sham, and significantly higher mean reduction from baseline in central retinal/subfield thickness (CRT; i.e., the 1-mm central macular subfield of the study eye, centred on the fovea [14]), with a favourable safety profile. Moreover, DEX implant was efficacious in treating persistent DME, anti-VEGF–resistant DME, and difficult-to-treat DME in vitrectomised eyes, with few injections [11, 12, 15–17].

There is evidence that baseline clinical parameters (e.g., vision, retina thickness, and DR severity) correlate with DME response to intravitreal anti-VEGF treatment and prognosis [18–23]. Identification of patients’ baseline characteristics that could predict treatment response to DEX implant may facilitate treatment selection, especially in patients nonresponsive to anti-VEGF therapy. This study evaluated associations between baseline characteristics and the time to first DME resolution after DEX intravitreal implant treatment.

## Methods

### MEAD study design

The MEAD study methodology has been described previously [14] and is summarised in the Supplementary Information.

### Post hoc analysis and statistical methods

This study’s primary outcome was to assess relationships between patients’ baseline characteristics and time to first DME resolution in patients randomised to DEX implant 0.7 mg or sham in the MEAD study. Secondary outcomes included the median time to first DME resolution per tertiles of baseline CRT (the 1-mm central macular subfield of the study eye, centred on the fovea [14]), and relationships between patients’ baseline factors and DME resolution rate at Month 36.

Data from the MEAD study were pooled. The full analysis set included all 351 patients treated with DEX implant 0.7 mg and 350 patients treated with sham procedure. Patients treated with DEX 0.35 mg (*N* = 347) were excluded as this formulation is not available for clinical use. DME resolution was defined as CRT < 250 μm by time-domain OCT during the study, based on DRCR.net recommendations for DME treatment [24]. The baseline factors studied were age, sex, race, ethnicity, diabetes type, diabetes duration, glycated haemoglobin (HbA1c), BCVA, CRT, DME duration and prior treatment, DME perfusion, DR severity (graded by the Early Treatment Diabetic Retinopathy Study [ETDRS] criteria), and lens status [25]. Eyes were also categorised in tertiles of baseline CRT.

Time to first DME resolution was analysed with the Kaplan-Meier method and log-rank tests. Univariate and multivariate Cox regression analyses were run to identify baseline factors predictive of time to first DME resolution. Lastly, univariate and multivariate logistic regressions were used to analyse the effect of baseline factors on DME resolution rate at Month 36. All analyses were in the intention-to-treat population with last observation carried forward for missing values.

Statistical analysis was performed with SAS version 9.4 (SAS Inc., Cary, North Carolina) with a 2-sided alpha level of 0.05.

## Results

Patients’ baseline characteristics, balanced between treatment groups, are summarised in Table 1. In the DEX implant and sham groups, respectively, mean age was 62.5 and 62.5 years, 60.7% and 62% of patients were male, 66.7% and 66.6% were Caucasian, 89.5% and 92% were type 2 diabetic, mean BCVA was 56.1 and 56.9 letters, mean CRT was 463.0 and 460.9 μm, and 49.3% and 49.7% had moderately severe nonproliferative DR or better.

**Table 1:** Demographics and baseline characteristics of patients and study eyes

	DEX Implant 0.7 mg (*N* = 351)	Sham (*N* = 350)	Total (*N* = 701)
Sex, *n* (%)			
Female	138 (39.3)	133 (38)	271 (38.7)
Male	213 (60.7)	217 (62)	430 (61.3)
Age (years), mean (SD)	62.5 (8.3)	62.5 (9.5)	62.5 (8.9)
Race, *n* (%)			
Caucasian	234 (66.7)	233 (66.6)	467 (66.6)
Asian	54 (15.4)	53 (15.1)	107 (15.3)
Hispanic	35 (10.0)	33 (9.4)	68 (9.7)
Black	16 (4.6)	20 (5.7)	36 (5.1)
Japanese	1 (0.3)	1 (0.3)	2 (0.3)
Other	11 (3.1)	10 (2.9)	21 (3)
Ethnicity, *n* (%)			
Caucasian	234 (66.7)	233 (66.6)	467 (66.6)
Non-Caucasian	117 (33.3)	117 (33.4)	234 (33.4)
Diabetes, *n* (%)			
Type 2	314 (89.5)	322 (92)	636 (90.7)
Type 1	34 (9.7)	28 (8)	62 (8.8)
Missing	3 (0.9)	0	3 (0.4)
Diabetes duration (years), mean (SD) *n*	16.5 (9.0) 349	15.9 (9.1) 348	16.2 (9.1) 697
HbA1c (%), mean (SD) *n*	7.58 (1.2) 349	7.50 (1.1) 349	7.54 (1.1) 696
DR severity, *n* (%)			
Moderately severe NPDR or better	173 (49.3)	174 (49.7)	347 (49.5)
Severe NPDR or worse	151 (43)	149 (42.6)	300 (42.8)
Missing	27 (7.7)	27 (7.7)	54 (7.7)
DME duration (months), mean (SD) *n*	23.6 (26.0) 350	25.9 (27.3) 349	24.7 (26.6) 699
DME perfusion, *n* (%)			
Ischaemic	43 (12.3)	27 (7.7)	70 (10.0)
Non-ischaemic	257 (73.2)	284 (81.1)	541 (77.2)
Missing	51 (14.5)	39 (11.1)	90 (12.8)
Prior DME treatment, *n* (%)			
Yes	247 (70.4)	261 (74.6)	508 (72.5)
No	104 (29.6)	89 (25.4)	193 (27.5)
BCVA (letters), mean (SD)	56.1 (9.9)	56.9 (8.7)	56.5 (9.3)
CRT (µm), mean (SD) *n*	463 (157.1) 348	460.9 (132.6) 342	462.0 (145.4) 690
Lens status, *n* (%)			
Phakic	265 (75.5)	249 (71.1)	514 (73.3)
Pseudophakic	86 (24.5)	101 (28.9)	187 (26.7)

*BCVA* best-corrected visual acuity, *CRT* central retinal thickness, *DEX* implant dexamethasone intravitreal implant, *DME* diabetic macular oedema, *DR* diabetic retinopathy, *HbA1c* haemoglobin A1c, *NPDR* nonproliferative diabetic retinopathy, *SD* standard deviation

**Fig. 1:**
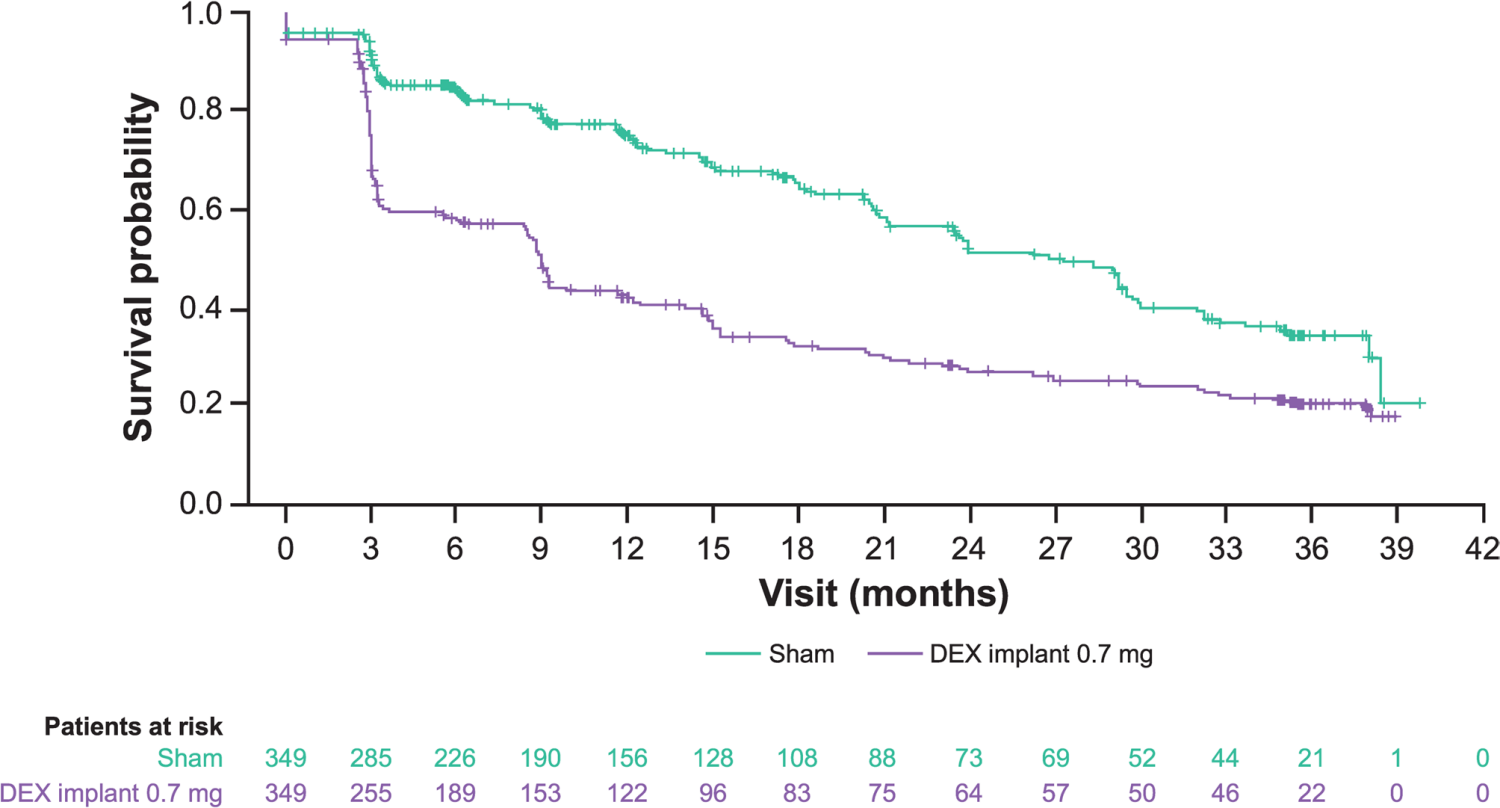
Kaplan-Meier curve of median time to first DME resolution after treatment initiation. *DEX* dexamethasone intravitreal implant, *DME* diabetic macular oedema

The survival analysis of time to first resolution of DME showed a median (95% confidence interval [CI]) time of 9.0 (8.5–9.3) months in DEX implant-treated patients versus 26.8 (21.2–29.5) months in sham-treated patients (Fig. 1). The estimate (95% CI) of nonresolution of DME at Month 36 was 20.1% (15.4–25.2%) in DEX implant-treated patients versus 34.1% (26.7–41.7%) in sham-treated patients. The hazard ratio (HR) for DME resolution (DEX implant versus sham) was 2.09 (*P* < 0.0001).

Baseline CRT had the largest univariate test statistics and was the most influential baseline characteristic on the median time to first DME resolution in both groups (DEX implant and sham). In DEX implant-treated patients, higher CRT (HR 1.00, *P* < 0.001) and increasing age (HR 0.98, *P* = 0.005) were associated with longer time to DME resolution, whereas DME ischaemia (HR 1.45, *P* = 0.046) and longer diabetes duration (HR 1.02, *P* = 0.023) were associated with shorter time to DME resolution. In sham-treated patients, higher CRT (HR 0.99, *P* < 0.001), increasing age (HR 0.98, *P* = 0.021), Caucasian race (HR 0.60, *P* = 0.003), and prior DME treatment (HR 0.63, *P* = 0.012) were associated with longer time to DME resolution, whereas DME ischaemia (HR 1.89, *P* = 0.046) was associated with shorter time to DME resolution (Table 2).

**Table 2:** Association of baseline characteristics with time to first DME resolution

	DEX Implant 0.7 mg			Sham		
	Estimate	HR	*P* Value	Estimate	HR	*P* Value
Sex	-0.075	0.93	0.564	0.145	1.16	0.412
Age	-0.022	0.98	**0.005**	-0.020	0.98	**0.021**
Race	-0.103	0.90	0.440	-0.519	0.60	**0.003**
Ethnicity	-0.103	0.90	0.440	-0.519	0.60	**0.003**
Diabetes type	-0.199	0.82	0.374	0.073	1.08	0.809
Diabetes duration	0.015	1.02	**0.023**	0.006	1.01	0.515
HbA1c	-0.064	0.94	0.264	0.079	1.08	0.329
DR severity	0.062	1.06	0.171	-0.013	0.99	0.818
DME duration	0.003	1.00	0.174	0.001	1.00	0.810
DME perfusion	0.370	1.45	**0.046**	0.636	1.89	**0.046**
Prior DME treatment	-0.121	0.89	0.391	-0.470	0.63	**0.012**
BCVA	-0.004	1.00	0.558	0.002	1.00	0.842
CRT	-0.002	1.00	**< 0.001**	-0.005	0.99	**< 0.001**
Lens status	-0.149	0.86	0.317	0.368	1.44	0.062

Results from Univariate Cox Regression analyses. Statistically significant *P* values are bolded

*BCVA* best-corrected visual acuity, *CRT* central retinal thickness, *DEX implant* dexamethasone intravitreal implant, *DME* diabetic macular oedema, *DR* diabetic retinopathy, *HbA1c* haemoglobin A1c, *HR* hazard ratio

Tertiles of increasing baseline CRT were associated with significantly longer median time to first DME resolution and lower cumulative incidence of DME resolution in sham-treated patients (median time of 15.2, 26.8, and 32.2 months for T1–T3, respectively), compared with DEX implant-treated patients (median time of 3.2, 9.2, and 14.9 months for T1–T3, respectively; HR of 1.9, 2.2, and 2.6 for T1–T3, respectively; all *P* < 0.0001).

In DEX implant-treated patients, higher CRT was significantly associated with nonresolution of DME at Month 36 (odds ratio [OR] [95% CI] 1.00 [1.00–1.00]; *P* = 0.005). In sham-treated patients, higher CRT was significantly associated with nonresolution of DME at Month 36 (OR 1.00 [0.99–1.00]; *P* < 0.0001), as well as increasing age (OR 0.97 [0.95–1.00]; *P* = 0.02), and Caucasian race (OR 0.44 [0.23–0.83]; *P* = 0.01) (Table 3).

**Table 3:** Association of baseline characteristics with DME resolution at month 36

	DEX Implant 0.7 mg					Sham				
	*n*	Responder *n* (%)	OR (95% CI)	*P* Value	C-Statistic	*n*	Responder *n* (%)	OR (95% CI)	*P* Value	C-Statistic
Sex										
Female	138	57 (41.3)			0.539	133	35 (26.3)			0.511
Male	211	71 (33.6)	0.72 (0.46–1.12)	0.147		216	61 (28.2)	1.10 (0.68–1.79)	0.695	
Age (years)	348	127 (36.5)	0.99 (0.96–1.01)	0.384	0.536	342	93 (27.2)	0.97 (0.95-1.00)	**0.027**	0.587
Race										
Asian	54	24 (44.4)			0.566	53	21 (39.6)			0.604
Caucasian	232	78 (33.6)	0.63 (0.35–1.16)	0.136		232	52 (22.4)	0.44 (0.23–0.83)	**0.010**	
Hispanic	35	18 (51.4)	1.32 (0.46–3.11)	0.519		33	16 (48.5)	1.43 (0.60–3.45)	0.420	
Black	16	5 (31.3)	0.57 (0.17–1.86)	0.350		20	5 (25.0)	0.51 (0.16–1.61)	0.249	
Japanese	1	0	0.00 (0.00, I)	0.986		1	0	0.00 (0.00, I)	0.987	
Other	11	3 (27.3)	0.47 (0.11–1.96)	0.299		10	2 (20.0)	0.38 (0.07–1.97)	0.250	
Ethnicity										
Caucasian	232	78 (33.6)			0.544	232	52 (22.4)			0.585
Non-Caucasian	117	50 (42.7)	1.47 (0.93–2.33)	0.096		117	44 (37.6)	2.09 (1.28–3.39)	**0.002**	
Diabetes type										
Type 1	34	10 (29.4)			0.516	28	9 (32.1)			0.509
Type 2	313	118 (37.7)	1.45 (0.67–3.14)	0.343		321	87 (27.1)	0.78 (0.34–1.80)	0.567	
Diabetes duration (years)	348	127 (36.5)	1.02 (1.00-1.05)	0.096	0.558	342	93 (27.2)	1.01 (0.99–1.04)	0.420	0.531
HbA1c (%)	348	127 (36.5)	0.84 (0.70–1.03)	0.088	0.560	342	93 (27.2)	1.09 (0.88–1.37)	0.428	0.528
DR severity	348	127 (36.5)	1.06 (0.91–1.24)	0.443	0.527	342	93 (27.2)	0.99 (0.85–1.16)	0.928	0.497
DME duration (months)	348	127 (36.5)	1.01 (1.00-1.01)	0.205	0.520	342	93 (27.2)	1.00 (0.99–1.01)	0.573	0.470
DME perfusion										
Ischaemic	43	19 (44.2)			0.518	27	8 (29.6)			0.504
Non-ischaemic	257	96 (37.4)	0.75 (0.39–1.45)	0.394		283	78 (27.6)	0.90 (0.38–2.15)	0.818	
Prior DME treatment										
Yes	247	89 (36.0)			0.510	260	66 (25.4)			0.540
No	102	39 (38.2)	1.10 (0.68–1.77)	0.697		89	30 (33.7)	1.49 (0.89–2.52)	0.130	
BCVA (letters)	348	127 (36.5)	1.00 (0.98–1.02)	0.830	0.490	342	93 (27.2)	1.01 (0.98–1.04)	0.469	0.525
CRT (µm)	348	127 (36.5)	1.00 (1.00–1.00)	**0.005**	0.593	342	93 (27.2)	1.00 (0.99-1.00)	**< 0.001**	0.653
Lens status										
Phakic	263	92 (35.0)			0.528	248	70 (28.7)			0.513
Pseudophakic	86	36 (41.9)	1.34 (0.81–2.20)	0.251		101	26 (25.7)	0.88 (0.52–1.49)	0.637	

Results from Univariate Logistic Regression analyses. Statistically significant *P* values are bolded

*BCVA* best-corrected visual acuity, *CI* confidence interval, *CRT* central retinal thickness, *DEX implant* dexamethasone intravitreal implant, *DME* diabetic macular oedema, *DR* diabetic retinopathy, *HbA1c* haemoglobin A1c, *OR* odds ratio

There was no significant association between time to DME resolution and sex, diabetes type, HbA1c level, DR severity, DME duration, BCVA, and lens status in either treatment group.

## Discussion

In this analysis, the median time to first DME resolution was 9 months (DEX implant) versus 26.8 months (sham). The DEX implant increased the DME resolution rate by 2.09 times by Month 36 (versus sham). Higher CRT and older age at baseline prolonged the time to DME resolution in both treatment groups, whereas DME ischaemia shortened it. In sham-treated patients, Caucasian race and prior DME treatment also prolonged the time to DME resolution. In DEX implant-treated patients, longer diabetes duration shortened the time to DME resolution. Higher CRT was associated with a lower DME resolution rate at Month 36 in both treatment groups, whereas older age and Caucasian race had the same association only in sham-treated patients.

Early (16 weeks) and sustained (32 weeks; 1 year) improvements (≥ 20% reduction) in central subfield thickness (CST/CRT) have been associated with significantly better visual outcomes in DME patients treated with ranibizumab, regardless of number of injections [26]. Similarly, eyes with persistent DME during the first 52 weeks of ranibizumab treatment were associated with lower long-term visual improvement [18]. Although similar analysis is not available for DME eyes treated with steroids, early visual response to DEX implant correlated with long-term visual improvement in DME (poor responders [< 5 letters] at 3 months had lower rates of BCVA change ≥ 10 letters at 18 months, compared with robust responders [≥ 10 letters]; *P* = 0.009) [27]. Additionally, real-world data of naïve (with shorter disease duration) versus previously treated DME eyes (with longer disease duration) has shown that significantly more naïve patients treated with DEX implant present BCVA gains ≥ 15, ≥10, and ≥ 5 letters, compared with previously treated eyes [28]. Time to DME resolution thus influences visual outcomes, and understanding how baseline patient and disease characteristics predict treatment response is paramount to personalised medicine.

Treatment with DEX implant was associated with a longer time to first DME resolution (median, 9 months) than anti-VEGF agents (aflibercept: median, 33 weeks or ∼ 7 months; ranibizumab: mean, 6 months) [21, 22]. However, these studies observed different populations and used different OCT devices to assess DME, making direct comparisons challenging.

Thicker retina and worse vision, often hallmarks of more severe oedema, have been set as strong predictors of DME response to anti-VEGF therapy. A post hoc analysis of VIVID and VISTA found higher baseline CST/CRT associated with longer time to and lower rate of DME resolution in both the aflibercept and laser/control groups, whereas better baseline BCVA had the same association only in the aflibercept group [21]. Likewise, a meta-analysis of RISE, RIDE, and Protocols I, S, and T demonstrated that higher baseline CST/CRT was associated with a longer time to DME resolution [22]. Baseline vision did not predict response to DEX implant or sham treatment in our study. Consistent with the literature, however, this study found that higher baseline CRT is associated with a longer time to and lower rate of DME resolution. One possible explanation is that more fluid takes longer to clear, even with treatment. More fluid may also indicate more severe microstructural changes, as well as uncontrolled diabetes or comorbidities that could influence treatment response.

While the association between higher baseline CRT and longer time to DME resolution in patients treated with anti-VEGF injections is well established, a subset of patients may not exhibit meaningful reductions in CRT despite repeated injections. The persistence of thickened retinas in anti-VEGF non-responders suggests a potential role for alternative mechanisms beyond VEGF-driven vascular leakage, such as chronic inflammation, which are more directly targeted by DEX implants. The finding that higher CRT prolonged the time to DME resolution in patients treated with DEX implants, though consistent with the general trend seen with anti-VEGF agents, underscore that DEX implant still provide a viable resolution pathway even in patients with persistent or recurrent oedema, which is consistent with the literature [29]. Moreover, the corticosteroid’s mechanism of action addressing inflammatory pathways rather than solely VEGF-mediated permeability, may be particularly relevant for these patients. Prospective studies with prolonged follow-up are warranted to determine the long-term effects of the treatment.

Patients’ demographics/characteristics evaluated herein have been established as prognosticators of visual outcomes in DME eyes treated with anti-VEGF agents. In a post hoc analysis of Protocol T, visual acuity reduction was 2.1 letters at 2 years (95% CI, -3.0, -1.2; *P* < 0.001) and 1.9 letters over 2 years (95% CI, -2.4, -1.3; *P* < 0.001) for every decade of participant age, regardless of the anti-VEGF agent used [30]. In our study, older age prolonged the time to DME resolution in both groups, and lowered the DME resolution rate in sham-treated patients only. Although this has not been observed in anti-VEGF–treated patients, age-related homeostasis dysregulation may impact DME clearance. Aging bas been associated with a state of chronic inflammation that is a risk factor for the development and progression of chronic diseases such as cardiovascular and neurodegenerative diseases [31]. Although this process is highly heterogeneous across individuals, it is possible that age-related pro-inflammatory state interferes with time to DME resolution. Additionally, vitreous liquefaction secondary to aging may interfere with resolution of DME by reducing structural support or promoting traction on the macular region [32, 33]. Vitreous liquefaction also alters vitreous viscosity and diffusion properties, which impact drug delivery and may contribute to the longer time to DME resolution observed in the DEX implant group [34, 35].

Moreover, Bressler et al. [30] found that African American participants had greater CST/CRT reductions (model estimate, -12.0; 95% CI, -37.0, 13.0; *P* = 0.02) among patients studied in Protocol T. Our study revealed that Caucasians had a prolonged time to and lower rate of DME resolution in the sham group only, whereas no association between race/ethnicity and time to DME resolution was found in prior anti-VEGF studies [21, 22]. The finding that DME takes longer to resolve in Caucasian patients appears counterintuitive, as DME is driven by inflammation mechanisms and there is evidence that African Americans have higher degrees of low-grade inflammation [36–38]. In fact, this population has a higher prevalence of type 2 diabetes, DR, and DME, all possibly related to inflammation [4, 39, 40]. Nonetheless, non-White populations are underrepresented in DME clinical trials, which may underpower analyses of outcomes by race and ethnicity [41, 42]. Further investigation is warranted.

Diabetic macular ischaemia, characterised by enlargement of the foveal avascular zone, has shown prognostic value for DR progression, DME development, and treatment response [43–45]. Per Lee et al. [44], poor response to anti-VEGF was associated with a larger deep capillary plexus/foveal avascular zone in DME eyes (0.87 [0.41] mm^2^), compared with good responders (0.57 [0.22] mm^2^; *P* < 0.001), but no significant association was seen with superficial capillary plexus ischaemia (*P* = 0.884 for vascular flow density; *P* = 0.954 for foveal avascular zone area). In contrast, this study revealed that presence of DME ischaemia shortened the time to DME resolution in both DEX implant and sham groups. This may be driven by treatment of inflammation rather than angiogenesis with DEX versus anti-VEGF agents, but the reason for this finding in the sham group remains to be elucidated. One possible explanation is the reduced capacity of ischaemic, non-perfused retinal tissue to sustain vascular leakage, which may limit the degree of further fluid accumulation and thus leading to faster apparent anatomical resolution regardless of treatment. In the DEX implant group, the corticosteroid’s anti-inflammatory effects may further accelerate this process by supressing residual low-grade inflammation. However, in the sham group, this finding is more difficult to explain and may be partly related to structural changes associated with chronic ischaemia. Specifically, progressive retinal atrophy resulting from chronic ischaemia might contribute to lower CRT readings that mimic oedema resolution but actually reflect underlying neuroretinal tissue loss. This atrophy-related thinning would be particularly relevant in advanced disease, and could bias interpretation of “DME resolution” on OCT. While DR severity stage was weakly associated with the total area of capillary loss and presence or absence of ischaemia both in DEX and sham groups in all timepoints of the MEAD study (Table S1), it did not confound the association of DME ischaemia found in this study. Nevertheless, it is important to acknowledge that the MEAD study evaluated DME ischaemia with fluorescein angiography, which does not capture the deep capillary plexus as fully as OCT angiography (OCTA). Future studies leveraging these newer imaging modalities could better differentiate true oedema resolution from structural atrophy and provide a better understanding of the role of ischaemia in DME resolution dynamics.

Our study showed that prior treatment prolonged the time to DME resolution only in the sham group, supporting DEX implant’s efficiency in treating non-naïve DME eyes. Such association was not explored in prior studies with anti-VEGF therapy [21, 22]. However, prior treatment has been linked to visual outcomes, as Bressler et al. [30] found an association between the absence of prior panretinal photocoagulation treatment and larger visual acuity gains in patients studied in Protocol T.

An association between longer diabetes duration and shorter time to DME resolution in the DEX implant group was also found in this study. It is well established that longer diabetes duration correlates with the development and progression of DR and DME, regardless of diabetes type [46, 47]. Talcott et al. [22] have found more severe DR at baseline was linked to shorter time to first DME resolution within 24 months of ranibizumab treatment. In individuals with longer diabetes duration, severe DR may have led to chronic retinal damage, atrophy, and fibrosis, reducing the extent of oedematous tissue and thus shortening the time to resolution. Although DR severity was not a predictor of time to DME resolution in our study, the post hoc nature of the analysis may have limited the power to detect this association. Additionally, retinal ischaemia, more common in severe DR, may have influenced DME resolution as discussed earlier. Prolonged diabetes may also alter inflammatory pathways, leading to faster oedema reduction with corticosteroid treatment. Chronic hyperglycaemia and low-grade inflammation can upregulate cytokines, pro-apoptotic molecules, and leukocytes that cause retina neurodegeneration and vascular damage, which is cumulative over time and contribute to persistent oedema [48]. DEX implants, through broad-spectrum anti-inflammatory effects, might suppress these pathways more effectively, accelerating oedema resolution in eyes with prolonged disease duration. Furthermore, the changes in the integrity of the retina in longstanding DME, such as disorganization of the retina layers, may facilitate faster collapse of oedematous spaces once the inflammatory component is controlled, again resulting in a more rapid CRT reduction. However, the exact mechanisms behind the association of longer diabetes duration and shorter DME resolution time remain unclear, warranting further investigation.

This study also explored the prognostic effect of multiple other baseline characteristics on the time to DME resolution. There was no significant association found with sex, lens status, diabetes type, and HbA1c level, consistent with prior studies [21, 22]. However, it is noteworthy that the MEAD study did not include participants with HbA1c levels above 10%; this could explain why an association between uncontrolled disease and time to DME resolution was not found.

Study strengths include the large population from the MEAD study, which followed stringent protocols and data collection. Nonetheless, our post hoc analysis might contain inherited bias or limitations. Future prospective analyses are essential to validate our results and potentially unveil more nuanced relationships between baseline characteristics and treatment outcomes. The use of time-domain OCT with a fixed CRT cut-off of < 250 μm for defining DME resolution could influence the comparability of this study’s findings with those from studies using spectral-domain OCT devices or alternative thresholds. Time-domain OCT has lower axial resolution than spectral-domain OCT devices, leading to variable estimation of retinal thickness which could affect the reported results. Additionally, the MEAD trial was conducted over a decade ago, and evaluated response to DEX given at ≥ 6-month intervals. Retreatment practices have since evolved, with DEX implant being reinjected every 3–5 months based on patient response and disease activity [28, 49]. Thus, time to DME resolution may have been underestimated here, limiting direct applicability of our resolution time estimates. Assessment of the aforementioned associations at 3 or 6 months (before reinjection) could thus have been informative as well. Other limitations include not accounting for patterns of increased retina thickness (presence and volume of intraretinal fluid and/or subretinal fluid), retina thickness fluctuation, and other OCT-derived biomarkers (including presence of hyperreflective foci), which may also be related to time to DME resolution and final anatomical and visual outcomes [44, 50, 51].

In conclusion, this analysis indicates that DEX implant shortened the time to and increased the rate of DME resolution (versus sham) in participants of the MEAD study. Higher baseline CRT and increasing age were associated with a prolonged time to DME resolution. Our observations highlight the importance of baseline patient and disease characteristics as predictors of treatment outcomes, paving the way for more personalised, effective treatment strategies.

## Electronic supplementary material

Below is the link to the electronic supplementary material.

Supplementary Material 1

## Data Availability

AbbVie is committed to responsible data sharing regarding the clinical trials we sponsor. This includes access to anonymised, individual, and trial-level data (analysis data sets), as well as other information (e.g., protocols, clinical study reports, or analysis plans), as long as the trials are not part of an ongoing or planned regulatory submission. This includes requests for clinical trial data for unlicensed products and indications.These clinical trial data can be requested by any qualified researchers who engage in rigorous, independent, scientific research, and will be provided following review and approval of a research proposal, Statistical Analysis Plan (SAP), and execution of a Data Sharing Agreement (DSA). Data requests can be submitted at any time after approval in the US and Europe and after acceptance of this manuscript for publication. The data will be accessible for 12 months, with possible extensions considered. For more information on the process or to submit a request, visit the following link: https://vivli.org/ourmember/abbvie/ then select “Home.”

## Abbreviations

BCVA: Best Corrected Visual Acuity
CI: Confidence Interval
CRT: Central Retinal Thickness
CST: Central Subfield Thickness
DEX: Dexamethasone
DM: Diabetes Mellitus
DME: Diabetic Macular Edema
DR: Diabetic Retinopathy
DRCR.net: Diabetic Retinopathy Clinical Research Network
ETDRS: Early Treatment Diabetic Retinopathy Study
HbA1c: Hemoglobin A1c
HR: Hazard Ratio
OCT: Optical Coherence Tomography
OR: Odds Ratio
VEGF: Vascular Endothelial Growth Factor
