# Supplementary material for: Baseline factors affecting diabetic macular oedema resolution after intravitreal dexamethasone implant treatment: post hoc analysis of the MEAD study
Source: BMC Ophthalmol. 2025 Jul 9;25:403. doi: 10.1186/s12886-025-04208-3 (PMC12243367; doi:10.1186/s12886-025-04208-3)
Supplement: Supplementary file 1 — Supplementary Material 1 [file 12886_2025_4208_MOESM1_ESM.docx]

**SUPPLEMENTARY INFORMATION**

**MEAD Study Design**

The MEAD study methodology has been described previously.^1^ In summary, it consisted of identically designed, multicentre, randomised, masked, sham-controlled, 3-year, phase 3 trials conducted to evaluate the efficacy and safety of DEX implant for the treatment of DME. The MEAD study adhered to the tenets of the Declaration of Helsinki, complied with the Health Insurance Portability and Accountability Act, and was approved by the institutional review board or independent ethics committee at each research site.^1^ Written informed consent was obtained from all patients. The studies are registered at ClinicalTrials.gov with the identifiers NCT00168337 and NCT00168389.

Adult patients with type 1 or type 2 DM with fovea-involving DME that have been previously treated with medical (intravitreal anti-VEGF or intravitreal steroid) or laser therapy (focal/grid laser), or those who were treatment-naïve and refused or would not benefit from laser treatment, had BCVA between 34 and 68 letters as measured by the Early Treatment Diabetic Retinopathy Study (ETDRS) method (20/200-20/50), and had CRT in the 1-mm central macular subfield ≥300 μm as measured by time-domain OCT (TD-OCT) (OCT2 or OCT3; Stratus OCT, Carl Zeiss Meditec Inc., Dublin, CA, USA) were eligible for enrolment. Patients with history of treatment with intravitreal anti-VEGF within 3 months of study entry, treatment with intravitreal triamcinolone within 6 months of study entry, and current or anticipated use of systemic steroids were excluded. One eye per patient was included in the study. In case both eyes were eligible, the eye with shorter duration of DME was selected as the study eye. The 1048 patients enrolled were randomised 1:1:1 to receive DEX implant 0.7 mg (*n*=351), DEX implant 0.35 mg (*n*=347), or sham procedure (*n*=350) and could be retreated no more often than every 6 months. Patients were seen every 1.5 months for the first year, and every 3 months during the second and third years. BCVA (ETDRS) was obtained at each study visit and CRT (TD-OCT) was assessed every 3 months. Fluorescein angiography, fundus photographs, and safety were assessed at each visit and at safety visits 1, 7, and 21 days post-injection. Patients were eligible for retreatment if there was evidence of residual oedema and there had been at least 6 months since the most recent study treatment. For 95% of study treatments, retreatment eligibility required CRT >225 μm by OCT and investigator’s confirmation that the treatment would not put the patient at significant risk. A protocol amendment eventually allowed retreatment eligibility of CRT >175 μm by OCT or evidence of residual oedema on TD-OCT. The primary endpoint was achievement of ≥15-letter improvement in BCVA from baseline at the end of the study in the intention-to-treat population with last observation carried forward for missing values.

**REFERENCE**

1. Boyer DS, Yoon YH, Belfort RJ, et al. Three-year, randomized, sham-controlled trial of dexamethasone intravitreal implant in patients with diabetic macular edema. *Ophthalmology*. 2014;121:1904-1914. <https://doi.org/10.1016/j.ophtha.2014.04.024>.
